# Stigmatizing Attitudes Toward COVID-19 Among Patients, Their Relatives and Healthy Residents in Zhangjiajie

**DOI:** 10.3389/fpubh.2022.808461

**Published:** 2022-06-02

**Authors:** Xinxin Chen, Zhenjiang Liao, Shucai Huang, Qiuping Huang, Shuhong Lin, Yifan Li, Tianli Shao, Ying Tang, Jingyue Hao, Jing Qi, Yi Cai, Mingming Wang, Hongxian Shen

**Affiliations:** ^1^National Clinical Research Center for Mental Disorders and Department of Psychiatry, The Second Xiangya Hospital of Central South University, Changsha, China; ^2^Institute of Mental Health of Central South University, Chinese National Technology Institute on Mental Disorders, Hunan Key Laboratory of Psychiatry and Mental Health, Hunan Medical Center for Mental Health, Changsha, China; ^3^Department of Psychiatry, The Fourth People's Hospital of Wuhu, Wuhu, China; ^4^Department of Psychiatry, Comorbid Somatic Diseases, Kangning Hospital of Shenzhen, Shenzhen, China; ^5^Department of Psychiatry, Brain Hospital of Hunan Province, Changsha, China; ^6^Clinical Nursing Teaching and Research Section, The Second Xiangya Hospital of Central South University, Changsha, China; ^7^Zhangjiajie People's Hospital Designated for COVID-19 Treatment, Zhangjiajie, China

**Keywords:** COVID-19, personal stigma, perceived stigma, social distance, Zhangjiajie

## Abstract

**Introduction:**

In July 2021, Zhangjiajie City became the new epicenter of the COVID-19 outbreak. Aside from the physical manifestations of COVID-19, patients are also victims of severe social stigmatization. Stigma affects not only COVID-19 patients or survivors, but also individuals associated with them. This study aims to describe and assess the COVID-19-related stigma between patients, their relatives, and healthy local residents.

**Methods:**

The study included 43 COVID-19 patients, 68 relatives, and 75 healthy residents from Zhangjiajie. Demographic data was collected, including gender, age, marital status, and educational level. Stigma attitudes toward COVID-19 were measured using the Stigma Scale and Social Distance Scale. Frequencies and percentages were described for each item of the scales, and differences among the three groups were examined using the chi-square test.

**Results:**

With regards to personal and perceived stigma, most participants agreed that patients with COVID-19 “could snap out of the problem” and that “they were dangerous.” For social distance, over 30% of participants from the three groups agreed with the item “unwillingness to marry into the family of someone with COVID-19.” In all groups, there were significant statistical differences in the belief that “the problem is not a real medical illness” and the desire to “spend the evening socializing.”

**Conclusion:**

Although the outbreak was well-contained in Zhangjiajie, stigmatizing attitudes toward COVID-19 and desire for social distance to such patients were common among patients, their relatives and healthy local residents. Our study's results suggest that public education, anti-stigma interventions, and policies are necessary for people living in Zhangjiajie in order to effectively curtail the spread of COVID-19 and provide a useful strategy for a tourist city like Zhangjiajie to recover sooner from economic decline.

## Introduction

On January 30, 2020, the World Health Organization (WHO) declared the outbreak of coronavirus disease 2019 (COVID-19) as a Public Health Emergency of International Concern (PHEIC), and a pandemic on March 11, 2020 (1). As of early September 2021, more than 220 million people were infected worldwide (2). Notably, COVID-19 patients or survivors may have common psychiatric comorbidities and related issues, especially stigma (3). The stigma associated with disease increases the burden on individuals and society (4), adversely affecting families or communities that may be associated with infected individuals (5).

Stigma can be defined as a negative attitude associated with an individual with prejudices and discriminatory traits (6). It consists of self-stigma (attitudes about oneself) and public stigma (attitudes from others) (7). Particularly in the field of infectious diseases, stigma is recognized as a global challenge (8). In addition to the symptoms and deficits that result from these diseases, individuals suffering from infectious diseases constantly struggle with discrimination toward them from others (9). Unlike HIV, which has coexisted with humans for years, people often have higher levels of HIV-related knowledge and are less likely to stigmatize patients with HIV (10).

Nevertheless, as an emerging infectious disease that is evolving in nature and has highly accessible transmission patterns (the delta virus can infect the general population without protection in 14 s, increasing the transmission rate by nearly 100% compared to older strains), COVID-19 is likely to result in more panic and stigma among individuals and communities (11). Recent findings show that a proportion of COVID-19 survivors and their family members have been rejected and discriminated against by neighbors, landlords, and even employers (12), causing great financial and mental damage. Certain sub-populations, such as suspected COVID-19 patients, individuals discharged from quarantine, and individuals who have returned from overseas, also experience stigma, including social exclusion, being insulted, and stereotyping (13). Individuals experiencing social stigma may feel humiliated and reduce their help-seeking behaviors or fear approaching their relatives and friends; thus, increasing the challenge for public health efforts to effectively combat COVID-19 (14). Additionally, as a defense against contracting the virus, people naturally alienate individuals who are potentially infected with COVID-19 (e.g., people who live in or have traveled to high-risk areas), which subsequently increases COVID-19-related stigma (15). While avoidance behavior effectively reduces the risk of exposure to COVID-19, it may also induce stigma to people (such as patients and their relatives), cities (such as Wuhan and Zhangjiajie), and related populations (such as residents living in high-risk areas) (16). This means that although social distancing policies are paramount to preventing the spread of COVID-19, it may also adversely affect the attitudes of individuals and communities toward people, which may ultimately lead to stigmatizing conditions or stigma (17).

COVID-19 patients are victims of severe social stigmatization during the acute phase of illness, quarantine, and recovery periods (18). Being labeled with terms such as “super-spreader” and “infected the community” further increased the agony of COVID-19 survivors (19). Patients with COVID-19 reported significant self-stigma, expected stigma from family, neighbors and society during hospitalization (20). Some articles and reviews of COVID-19-related stigma have been published. For example, Duan et al. (4) found that 16% of Hubei province (the first epicenter of COVID-19 in China) residents reported feeling stigmatized, ashamed, or shuned and blamed themselves during the peak of the epidemic. Another study from Vietnam found that over 18% of medical workers felt worried about working in healthcare facilities after being quarantined, 10% felt condemned by relatives and friends, and 34% avoided contact with neighbors or others in their community (21). To date, a large number of studies have focused on COVID-19-related stigma among healthcare workers, patients, and survivors (4, 13, 21). However, there is a lack of research that focused on COVID-19-related stigma among inpatients, their relatives, and uninfected peers. One study revealed that healthy or uninfected individuals scored higher than COVID-19 survivors on stigma, social exclusion, financial insecurity, internalized shame, and social isolation (12), and the results of comparisons with relatives are still unknown. In this context, stigma affects not only individuals with stigmatizing attributes (e.g., COVID-19 patients or survivors) but also those associated with them (e.g., relatives, service providers, neighbors, and community members). Our study will assess COVID-19-related stigma among patients, their relatives, and uninfected individuals within close proximity in order to explore their differences.

Zhangjiajie is a world-famous city that attracts hundreds of millions of tourists every summer, making tourism the main source of the local economy. In July 2021, Zhangjiajie City suddenly became the new epicenter of the COVID-19 outbreak due to imported cases from other regions, resulting in more than 200 people affected with COVID-19. In an effort to curtail the spread of COVID-19, the local government declared an emergency lockdown; although measures have effectively contained the outbreak, the lockdown severely damaged the local economy. In this context, it is reasonable to assume that people with COVID-19 and their relatives experience more stigma than their uninfected peers because of the huge economic damage caused by the lockdown.

The aims of this study are twofold. First, to describe the COVID-19-related stigma during the COVID-19 outbreak in Zhangjiajie City; and second, to assess the difference in COVID-19-related stigma between patients, their relatives, and healthy local residents. Ultimately, we hope findings from our research will benefit policy making, patient rehabilitation, easing public panic, and providing guidance for targeted psychological interventions.

## Materials and Methods

### Participants

This study was an anonymous survey conducted from August 15 to 31, 2021, in Zhangjiajie, China. Participants diagnosed with COVID-19 were recruited from a designated COVID-19 hospital (Zhangjiajie City People's Hospital). Among the 72 patients with COVID-19, 22 critically ill patients (patients requiring a ventilator) and six under the age of 14 years were excluded. One patient was also excluded because the patient chose the same option for all questions; thus, only 43 patients were included. This study also included relatives of patients (*n* = 68) in the designated hospital. Additionally, Zhangjiajie' natives (*n* = 75) without COVID-19 were included as healthy controls through online survey.

The study protocol was approved by the Ethics Committee of the Second Xiangya Hospital of Central South University. All participants answered a yes/no question, indicating that they voluntarily participated in the study before completing the questionnaire.

### Measures

The questionnaires of the three groups were collected separately with three QR codes through “Questionnaire Star Platform,” a widely used Chinese questionnaire survey website. Demographic data included gender, age, marital status, and educational level.

#### Personal and Perceived Stigma Scale

Participants' stigma attitudes toward COVID-19 were measured using the stigma scale comprising of two subscales, personal stigma and perceived stigma, with each subscale including nine items (5). The items for both subscales contained identical items by asking participants' own attitudes and their views on people's attitudes toward patients diagnosed with COVID-19. In the present survey, the vignette was adapted as follows: “Susan is a person with COVID-19, the virus she was infected with was Delta.” A five-point scale ranging from “strongly agree” to “strongly disagree” was used to assess responses to each question, with higher scores indicating higher stigma. The Chinese stigma scale has good reliability and validity in assessing stigma toward several diseases in the Chinese population (22). In this study, Cronbach's α coefficients for the two subscales were 0.640 and 0.687, respectively.

#### Social Distance Scale

Participants' willingness to come into contact with people recovered from COVID-19 was estimated using the social distance scale (23). The scale contains five items, and all items are rated on a 4-point rating scale (1 =“definitely willing” to 4 = “definitely unwilling”), with higher mean scores indicating a higher tendency to social distance. The Chinese Social Distance Scale has good reliability and validity in assessing social distancing behavior in the Chinese population (22). In this study, Cronbach's α coefficient for the social distance scale was 0.924.

### Statistical Analysis

Demographic characteristics were described using median (interquartile range) and frequency (percentage). The chi-square (χ^2^) test and Kruskal-Wallis H tests were used to compare the differences in demographic data among the three groups. For each item of the stigma scale group, the percentage of “agree” or “strongly agree” category was listed. For items on social distancing, the percentage of “probably unwilling” or “definitely unwilling” category was listed in each group. Frequencies and percentages were described for each item of the stigma and social distance scale, and differences among the three groups. The personal and perceived stigma were examined by performing χ^2^ tests, and Bonferroni correction was used in the *post-hoc* analysis. Detailed data are shown in Tables 1–**3**. The significance level was set at *P* < 0.05, and all statistical analyses were conducted using IBM SPSS Statistics version 25.0.

**Table 1:** Demographic characteristics of participants.

**Variables**	**Total *n* = 186** **(n, %)**	**Patients *n* = 43 (*n*, %)**	**Relatives *n* = 68 (*n*, %)**	**Healthy people *n* = 75 (*n*, %)**	**Chi square**	*p*-value^*^
**Gender**					2.777	0.249
Male	67 (36.0%)	16 (37.2%)	29 (42.6%)	22 (29.3%)		
Female	119 (64.0%)	27 (62.8%)	39 (57.4%)	53 (70.7%)		
**Age (years)** ^**#**^	36 (26, 47)	35 (20, 52)	35 (27.25,46)	37 (27, 47)	0.344	0.842
**Marriage**					0.672	0.715
Married	128 (68.8%)	28 (65.1%)	46 (67.6%)	54 (72%)		
Unmarried and others (divorced, widowed)	58 (31.2%)	15 (34.9%)	22 (32.4%)	21 (28%)		
**Education**					26.216	<0.001
Below associate's degree	87 (46.8%)	33 (76.7%)	33 (48.5%)	21 (28%)		
Associate's degree and above	99 (53.2%)	10 (23.3%)	35 (51.5%)	54 (72%)		

* *P-value was calculated, with Chi-square test for categorical variables and Kruskal-Wallis test for continuous variables*.

# *Data descriptive with median (interquartile range)*.

## Results

A total of 186 participants from Zhangjiajie were included in the current study, with 43 patients, 68 relatives, and 75 healthy individuals. In the sample of patients, 37.2% were male and the average age was 36.79 years (range: 14–70, standard deviation [SD] = 16.345). For relatives, 42.6% were male and the average age was 35.75 years (range:15–77, SD = 12.468). Among the healthy individuals, 29.3% were male and the mean age was 36.84 years (range:14–59, SD = 11.436). There were no significant differences among these three groups for each sociodemographic variable (*p* > 0.05), except for education (*p* < 0.001). Details of the demographic characteristics are showed in Table 1.

### Personal Stigma

The three groups' attitudes toward the person in the vignette are shown in Table 2. Participants were more likely to endorse the item “the person could snap out of the problem.” This was particularly notable in patients and relatives, as over 88.0% agreed or strongly agreed with the statement. Next was the item “people with this problem are dangerous” where over 60% of participants agreed or strongly agreed with this representation, especially in patients (69.8%) and healthy people (68.0%). Endorsement of the personal stigma items that “the person could snap out of the problem” (88.4%) and that “the problem is a sign of personal weakness” (44.2%), “people with this problem are dangerous” (69.8%), and “people with this problem are unpredictable” (23.3%) were highest in patients. Beliefs that “the problem is not a real medical illness” (69.1%), “avoid people with this problem” (7.4%), “If I had this problem, I would not tell anyone” (14.7%), “I would not employ someone with this problem” (11.8%), and “I would not vote for a politician with this problem” (22.1%) were highest in relatives. Participants were least likely to endorse the statement “avoid people with this problem”; only 4.7% of patients, 7.4% of relatives, and 2.7% of healthy people expressed their agreement with this item. There were significant differences among these three groups in one statement, “the problem is not a real medical illness” (*p* = 0.007).

**Table 2:** Compared on each statement about participants' own attitudes toward the person in vignette.

**Statement about personal stigma**	**Patients (*****n*** **= 49)**		**Relatives (*****n*** **= 68)**		**Healthy people (*****n*** **= 75)**		**Total (*****n*** **= 186)**	
	***n***	**%**	***n***	**%**	***n***	**%**	***n***	**%**
1. The person could snap out of the problem	38	88.4	60	88.2	61	81.3	159	85.5
2. Problem is a sign of personal weakness	19	44.2	30	44.1	29	38.7	78	41.9
3. Problem is not a real medical illness^*^	18b	41.9	47a,c	69.1	36b	48.0	101	54.3
4. People with this problem are dangerous	30	69.8	37	54.4	51	68.0	118	63.4
5. Avoid people with this problem	2	4.7	5	7.4	2	2.7	9	4.8
6. People with this problem are unpredictable	10	23.3	13	19.1	10	13.3	33	17.7
7. If I had this problem, I wouldn't tell anyone	4	9.3	10	14.7	7	9.3	21	11.3
8. I would not employ someone with this problem	5	11.6	8	11.8	7	9.3	20	10.8
9. I would not vote for a politician with this problem	7	16.3	15	22.1	12	16.0	34	18.3

*Symbols flagging table entries denote significant differences relative to ^a^patients, ^b^relatives, ^c^healthy people*.

* *p < 0.05*.

### Perceived Stigma

The agreements of the three groups regarding other people's attitudes toward the person in the vignette are shown in Table 3. Consistent with personal stigma, participants were most likely to agree with the item “the person could snap out of the problem” and “people with this problem are dangerous” with 79 and 49.5% in total, respectively. Endorsement of people with this problem as “dangerous” (69.8%), and “unpredictable” (23.3%) were the highest in patients. Beliefs that “the person could snap out of the problem” (82.7%), “I would not tell anyone” (22.7%), and “I would not employ someone with this problem” (22.7%) were highest in healthy people. Agreement of “the problem is a sign of personal weakness” (50%), “the problem is not a real medical illness” (54.4%), “avoid people with this problem” (17.6%), and “I would not vote for a politician with this problem” (23.5%) were the highest among relatives. Participants were least likely to agree with the statement “Avoid people with this problem”; only 11.6% of patients, 17.6% of relatives, and 9.3% of healthy people expressed their agreement on this item.

**Table 3:** Compared on each statement about other people's attitudes toward the person in vignette.

**Statement about perceived stigma**	**Patients (*****n*** **= 49)**		**Relatives (*****n*** **= 68)**		**Healthy people (*****n*** **= 75)**		**Total (*****n*** **= 186)**	
	***n***	**%**	***n***	**%**	***n***	**%**	***n***	**%**
1. The person could snap out of the problem	33	76.7	52	76.5	62	82.7	147	79.0
2. Problem is a sign of personal weakness	14	32.6	34	50.0	32	42.7	80	43.0
3. Problem is not a real medical illness	19	44.2	37	54.4	34	45.3	90	48.4
4. People with this problem are dangerous	25	58.1	28	41.2	39	52.0	92	49.5
5. Avoid people with this problem	5	11.6	12	17.6	7	9.3	24	12.9
6. People with this problem are unpredictable	12	27.9	17	25.0	19	25.3	48	25.8
7. If I had this problem, I wouldn't tell anyone	5	11.6	10	14.7	17	22.7	32	17.2
8. I would not employ someone with this problem	6	14.0	15	22.1	17	22.7	38	20.4
9. I would not vote for a politician with this problem	8	18.6	16	23.5	17	22.7	41	22.0

### Social Distance

The endorsement of the three groups for willingness to contact Susan in the vignette are described in Table 4. Over 30% of participants expressed unwillingness to marry into the family of someone with COVID-19, with 27.9, 29.4, and 41.3% in patients, relatives, and healthy people, respectively. Healthy people endorsed the highest in four of the five items in social distance, and were more likely to avoid a person with COVID-19. There were significant differences among the three groups on this statement, “spend the evening socializing” (*p* = 0.042).

**Table 4:** Compared on each statement about social distance toward the person in vignette.

**Social interactions**	**Patients (*****n*** **= 49)**		**Relatives (*****n*** **= 68)**		**Healthy people (*****n*** **= 75)**		**Total (*****n*** **= 186)**	
	***n***	**%**	***n***	**%**	***n***	**%**	***n***	**%**
Live next door	4	9.3	4	5.9	11	14.7	19	10.2
Spend the evening socializing^*^	4	9.3	3c	4.4	13b	17.3	20	10.8
Make friends	7	16.3	5	7.4	10	13.3	22	11.8
Work closely	7	16.3	9	13.2	17	22.7	33	17.7
Marry into family	12	27.9	20	29.4	31	41.3	63	33.9

*Symbols flagging table entries denote significant differences relative to ^a^patients, ^b^relatives, ^c^healthy people*.

* *p < 0.05*.

## Discussion

To the best of our knowledge, this is the first study to investigate stigma among COVID-19 patients, their relatives, and healthy individuals at the same time. Furthermore, we concentrated our samples in Zhangjiajie, a tourism city in the west of Hunan province, which suddenly suffered from a COVID-19 outbreak in August 2021. The survey showed that participants were most likely to agree with the statements “the person could snap out of the problem” and “people with this problem are dangerous.” The statement most frequently attributed with unwillingness for participants was marrying into the family of someone with COVID-19. These findings are similar to those of previous studies of other diseases, such as schizophrenia, depression, and generalized anxiety disorder (7, 24).

Most participants would agree that patients with COVID-19 could snap out of the problem and that they were dangerous. It is noteworthy that the item “the person could snap out of the problem” did not seem appropriate for COVID-19. In this study, the item may indicate that people are confident they have control over the prognosis of the disease. Moreover, although beliefs about dangerousness are conducive to enhancing physical distance and preventing the spread of infection (25), this may also lead to higher stigma toward patients with COVID-19, which adversely affects their work and daily lives, even after recovery. Therefore, to effectively curtail the spread of the disease, it is necessary not only to maintain physical distance, but also educate and reduce the public's fear of COVID-19.

In the present study, perceived stigma received a much higher endorsement than personal stigma in avoidance of someone with this problem, unpredictability, and unwillingness to hire someone with this problem. Participants were more likely to believe that others held stigmatizing attitudes toward COVID-19 survivors. This indicates that stigma toward COVID-19 may be overestimated by the public (26). Similar to a study that explored mental disorders, more participants agreed with a perceived stigma in order to be socially accepted (24).

Our findings are also consistent with previous studies, which found that social distance was highest for marrying into the family for all three groups (22). From the first item “Live next door” to the last item “Marry into family,” the proportion of agreement increased in turn, even if <5% of the participants endorsed the item that they would avoid people with this problem in the personal stigma item. This may be because the desire for social interaction decreases with the level of intimacy (27, 28).

The results did not show significant differences in personal and perceived stigma among the three groups except for one statement, “the problem is not a real medical illness.” As for social distance, only one statement, “spend the evening socializing” had a significant difference among the three groups. This was slightly different from the results of previous studies. A Chinese study found that COVID-19 survivors experienced more severe COVID-19 related stigma compared with healthy controls (12). A high level of stigma might be due to concerns transmitted to family members and friends, as well as fear of being discriminated (12, 29).

Previous studies also showed that increased contact with individuals suffering from a disease (such as friends and family members) and higher knowledge of a disease predict lower stigma and social distance (30). This means that patients and family members may report lower levels of stigma and social distance. However, there were few significant differences in stigma and social distance among different populations in this study, which might be due to the well-controlled outbreak and the efforts made to popularize public health education on COVID-19-related knowledge in China.

This study presents several limitations. First, although the instrument has been widely validated in previous studies of the Chinese population, it has rarely been used in COVID-19-related research. Second, healthy control group was a convenient sample and may not have been representative of all residents. Furthermore, the participants educational level did not match the other two groups, which may cause some bias for unpaired COVID-19 related knowledge. Finally, since COVID-19 was effectively controlled in Zhangjiajie and did not become widespread, the sample size of patients and relatives was relatively small in this study. Future studies of stigma attitudes toward COVID-19 patients and survivors should be carried out in a larger sample with more appropriate tools.

## Conclusion

In conclusion, our survey demonstrated that COVID-19-related stigma is commonly experienced by COVID-19 patients, their relatives, and healthy residents even though the outbreak has been well-contained in Zhangjiajie City. Given the severe damage caused by COVID-19-related stigma, routine assessment of stigma should be conducted in each group, including patients or survivors, relatives, healthy individuals, and medical workers. Sufficient psychological assistance should be provided to those have suffered from these experiences. Additionally, improved identification, prevention, and policy effort are needed to reduce stigmatizing responses by social institutions and the general public. We hope that our survey, which focused on Zhangjiajie City, can have an impact on or aid similar cities that typically receive influxes of tourists, and as the world slowly eases border restrictions and leisurely travel becomes more common, it can be expected that the same situation will occur in other cities. Finally, prospective study designs should be used in future studies to examine the long-term impact of COVID-19-stigma.

## Data Availability Statement

The raw data supporting the conclusions of this article will be made available by the authors, without undue reservation.

## Ethics Statement

The studies involving human participants were reviewed and approved by Ethics Committee of the Second Xiangya Hospital of Central South University. Written informed consent to participate in this study was provided by the participants' legal guardian/next of kin.

## Author Contributions

HS and MW conceptualized and designed the research. SH, SL, and YL prepared the assessment tools. TS and YT performed the experiments. YC, JQ, and JH undertook the statistical analysis. QH wrote the first draft of the manuscript. XC and ZL contributed to the final manuscript. All authors approved the final version for publication and made substantial contributions to this study.

## Funding

This work was supported by the National Natural Science Foundation of China (No. 81971249), National Key R&D Program of China (No. 2020YFC2005300), Natural Science Foundation of Hunan Province (No. 2020JJ4782), and Hunan Development and Reform Commission [2019] No.412 Innovation R&D Project.

## Conflict of Interest

The authors declare that the research was conducted in the absence of any commercial or financial relationships that could be construed as a potential conflict of interest.

## Publisher's Note

All claims expressed in this article are solely those of the authors and do not necessarily represent those of their affiliated organizations, or those of the publisher, the editors and the reviewers. Any product that may be evaluated in this article, or claim that may be made by its manufacturer, is not guaranteed or endorsed by the publisher.
